# Analgesic, Anti-inflammatory and *In-vitro* Hyaluronidase Inhibitory Properties of the Leaf Extract and Solvent Fractions of *Otostegia Fruticosa* (Forssk.) Schweinf. ex Penzig.

**DOI:** 10.22037/ijpr.2019.14657.12569

**Published:** 2020

**Authors:** Tiegsti Bahta, Aman Karim, Gomathi Periasamy, Gereziher Gebremedhin, Najeeb ur-Rehman, Helen Bitew, Kalay Hagazi

**Affiliations:** a *Department of Pharmacognosy, School of Pharmacy, College of Health Sciences, P.O Box 1871, Mekelle University, Mekelle, Ethiopia. *; b *Department of Pharmacy, College of Health Sciences, Aksum University, P.O Box 298, Aksum, Ethiopia. *; c *Department of Pharmacology, College of Pharmacy, Prince Sattam Bin Abdulaziz University, Al-Kharj 11942, Saudi Arabia.*

**Keywords:** Analgesic activity, Anti-inflammatory activity, Otostegia fruticose, Tail immersion test, Acetic acid- induced writhing, Hyaluronidase inhibition

## Abstract

*Otostegia fruticosa* is traditionally used to treat tonsillitis, stomach ache, asthma, arthritis, and febrile illness in different parts of Ethiopia and other countries. In this experiment 70% ethanolic crude extract and fractions of the leaf of *Otostegia fruticosa* (Forssk.) Schweinf. ex Penzig were evaluated for their *in-vivo *anti-inflammatory and analgesic activities and *in-vitro *hyaluronidase inhibition properties at different concentrations. Tail immersion, acetic acid induced writhing and carrageenan-induced paw edema model were used to assess the *in-vivo* analgesic and anti-inflammatory activities, respectively. Swiss albino mice of either sex were randomly divided into five groups of six mice per group and for evaluation of the fractions randomly divided into six groups of six mice per group. The test groups were treated with hydroalcoholic extract of *Otostegia fruticosa (O. fruticosa) *at doses of 100, 200, and 400 mg/kg. The positive control groups received either pethidine 5 mg/kg or aspirin at 100 mg/kg or 150 mg/kg. The negative control groups were orally given sunflower oil. All the fractions were administered at the dose of 400 mg/kg. In all models, the higher dose (400 mg/kg) of the crude extract and chloroform fraction showed a significant central and peripheral analgesic and anti-inflammatory activities with comparable effects to standards used. The hyaluronidase inhibition assay result showed that the test samples displayed concentration-dependent inhibitory activities. These findings indicate that 70% ethanol extract and organic solvent fractions of *O. fruticosa* leaves have potential analgesic, anti-inflammatory, and enzyme inhibitory activities.

## Introduction

The genus *Otostegia* contains about 33 species under the family Lamiaceae which are endemic to the northern part of tropical Africa and South Western and Central Asia (1). *Otostegia fruticosa* (Forssk.) Schweinf. ex Penzig is a shrub widely distributed in Ethiopia, Eritrea, Djibouti, Sudan, Cameroon, Saudi Arabia, Yemen, Israel, Sinai, and Palestine. The branches are more or less densely hairy; the leaves are oval to rounded and are 5-12 cm long. The flowers are cream in colour. *O. fruticosa*is commonly called by its vernacular name “sasa or geram tungut” in Ethiopia (2, 3) and has been used in the treatment of tonsillitis, stomach ache, asthma, arthritis, febrile illness, sun-stroke, and gynaecological problems.

The International Association for the Study of Pain (IASP) defined pain as “an unpleasant sensory and emotional experience associated with actual or potential tissue damage or described in terms of such damage” (4). Pain is a vast worldwide public health problem and estimates suggest that 20% of adults suffer from pain globally and 10% are newly diagnosed with chronic pain each year (5). Inflammation is a body defense reaction in order to eliminate or limit the spread of injurious agents as well as to remove the consequent necrosed cells and tissue (6). It is associated with alteration of signalling pathways which results in the increased levels of inflammatory markers, lipid peroxides, and free radicals (7). The results of each inflammatory reaction may be beneficial (defend the body against agents deranging its homeostasis) or harmful (damage to surrounding tissues) (6).

NSAIDs are used globally for the treatment of inflammation, pain, and fever (8). These drugs have serious limitations because of their side effects such as gastric irritation and gastric ulcer, alterations in renal function, effects on blood pressure, hepatic injury and platelet inhibition which may result in increased bleeding and dependency (9). The risk of mortality as a result of chronic use of NSAIDs is 1 in 10,000 for young adults aged 16-45 and it increases tenfold for those over 75 years old. In addition, to the side effects, synthetic drugs are very costly to develop (10).

Natural products and plant based drugs in traditional medicine are being paid much courtesy due to their least side effects, cheapness, and the fact that majority of the world population in developing countries still rely on them (11). Unlike the conventional drugs which are single active component that target one specific pathway, herbal medicines work in a way that hinges on an orchestral approach. The research and analysis of the plants used in relieving pain and inflammatory conditions in traditional ethnomedicine are one of the productive and logical approaches in the search for new drugs (12, 13). In this study; the *in-vivo* anti-inflammatory, central and peripheral analgesic activities and *in-vitro* hyaluronidase inhibition properties of the leaves extract of *O. fruticosa *have been evaluated.

## Experimental

*Chemicals and drugs*

The following chemicals, solvents, reagents, and drugs were used: Normal saline (Freseniu kabi, India), petroleum ether (Blulux Laboratories Ltd., India), HCl (Fisher Chemicals, UK), potassium ferrocyanide (Blulux Laboratories Ltd., India), lead acetate (Blulux Laboratories Ltd., India), chloroform (Carlo ERBA Reagents SAS, France), ferric chloride (Blulux Laboratories Ltd., India), absolute methanol and ethanol (Carlo ERBA Reagents SAS, France), ethyl acetate (Carlo ERBA Reagents SAS, France), acetic anhydride (Blulux Laboratories Ltd, India), n-butanol (Carlo ERBA reagents SAS, France), hexane (Laboratory Fine Chemicals Pvt. Ltd. India), acetylsalicylic acid and pethidine (Julphar Pharmaceuticals Ethiopia), carrageenan (Sigma-Aldrich Steinheim, Germany), glacial acetic acid (Loba Chemicals, India), and sunflower oil.

*Plant material*

The leaves of *O. fruticosa* were collected in January 2017 from Wukro Kilteawlaelo 45 Km east of Mekelle, Northern Ethiopia. The collected plant specimen was identified and authenticated by a botanist Mr. Shamble Alemu and a voucher specimen of the plant (001) was deposited at the National Herbarium of College of Natural and Computational Sciences, Addis Ababa University.

*Experimental animals*

Swiss albino mice of either sex weighing 25-35 g and age 6-8 weeks were obtained from the animal house of the School of Pharmacy, Mekelle University. The mice were kept at room temperature with a 12 h light/dark cycle, food and water *ad libitum*. They were acclimatized for a week before commencement of the experiment. All the experiments were conducted in accordance with the internationally accepted laboratory animal use and care guideline for (14).

*Preparation of crude extract*

The leaves were air-dried under shade and then ground into coarse powder using mortar and pestle. The coarse powder was packed in plastic bags and stored in dry and well-ventilated room. The powdered plant material (800 g) was soaked in 6.4 L of 70% ethanol; and placed on an orbital shaker at 130 rotations per minute (rpm). The extract was filtered after 72 h, using muslin fabric followed by Whatman filter paper No 1. The residue was re-macerated twice to exhaustively extract the plant material. The filtrates were combined and dried using a drying oven at 40 °C. Finally, the dried extract was collected in a well-closed glass bottle covered with aluminium foil and stored in a refrigerator at -4 °C until next use.

*Acute oral toxicity test*

Acute oral toxicity study was conducted as per the internationally accepted protocol drawn under the Organization for Economic Co-operation and Development guidelines 425 (15). Nulliparous female Swiss albino mice were used. Before oral administration of a single dose of the test samples, the mice were deprived from food for 3 h. Initially a single female mouse was given 2000 mg/kg of the extract orally. After 24 h following the results from the alive first mouse, other four female mice were administered a single dose of 2000 mg/kg. The mice were observed continuously for the first 30 min, after administration of the test sample; intermittently for 4 h over a period of 24 h and for 14 days. The weight of all animals before and after fasting, at 6^th^ and 14^th^ day was recorded. Gross behavioral changes such as loss of appetite, hair erection, lacrimation, tremors, convulsions, salivation, diarrhea, mortality, and other signs of toxicity manifestation were observed (15).

*Fractionation of crude extract*

The crude extract was further fractionated using the modified Kupchan method (16). The dried hydroalcoholic leaf extract of *O. fruticosa* (65 g) was dissolved in 90% methanol and successively partitioned using different solvents of increasing polarity (hexane, chloroform, ethyl acetate and n-butanol) in a separatory funnel. The different solvent fractions were concentrated and dried in an oven at a temperature not exceeding 40 °C. The dried fractions were then transferred into separate vials and stored in a fridge for further use.

*In-vivo analgesic and anti-inflammatory activities*

*Grouping of animals and dosing*

For testing the analgesic and anti-inflammatory activity of the crude extract, group I served as a negative control and was administered the vehicle sunflower oil (17). Groups II, III and IV were given 100, 200, and 400 mg/kg of the extract respectively and group V was administered standard drug *i.e.* 100 mg/kg acetylsalicylic acid for carrageenan-induced paw edema (18) and 150 mg/kg for acetic acid-induced writhing (19) and 5 mg/kg of pethidine (20) for tail immersion test. Similarly; for testing the analgesic and anti-inflammatory activity of the fractions groups II, III, IV, and V were given 400 mg/kg of chloroform, ethyl acetate, butanol, and hydro methanol fraction. 

*Tail immersion method*

In this study, analgesia was assessed by tail flick latency difference (TFLD) *i.e.* latency of mice to remove its tail clearly out of water at 51 °C. Mice were held in hand with only tail extending out. Then one third (2-3 cm) of the tail was submerged in a thermostatically controlled water bath maintained at 51 °C. The time in second taken to withdraw the tail totally out of the water was noted as the reaction time or tail-flick latency (21). The maximum cutoff time for immersion was 15 s in order to avoid injury of the tail tissues (22). The animals were subjected to the same test procedure at 0 (before) and 30, 60, 90, 120,150, and 180 min after treatment as described in the grouping and dosing section. The criterion for analgesia was post-drug latency which was greater than two times the pre-drug average latency. TFLD or mean increase in latency after drug administration was used to indicate the analgesia produced by test and standard drugs. Analgesia TFLD was calculated as follows (21).

Analgesia TFLD = 

Post-drug tail flick latency – Pre-drug tail flick latency

*Acetic acid-induced writhing test*

Writhing syndrome was elicited by intraperitoneal injection of 0.6% aqueous acetic acid (10 mL/kg). Number of writhing movements consisting of contraction of the abdominal muscles, drawing up of hind limbs toward the abdominal walls, stretching of hind limbs and periodic arching of the body displayed were counted for 20 min after a latency period of 5 min. The extracts and reference standard 150 mg/kg of acetylsalicylic acid (19) was administered in their respective doses 30 min prior to the test and percentage inhibition of writhing was calculated as follows (23).

$\mathrm{Inhibition}\left(\%\right)=\frac{\mathrm{Mean number of writhes}(\mathrm{control})-\mathrm{Mean number of writhes}(\mathrm{test})}{\mathrm{Mean number of writhes}(\mathrm{control})}\times 100$

*Carrageenan-induced paw edema test*

Following one hour after administration of vehicle, extracts and standard acute inflammation was produced by subplantar injection of carrageenan (0.05 mL of 1% w/v suspension), in the right hind paw of the mice. Inflammation was quantitated in terms of volume *i.e.* displacement of water by edema using a digital plethysmometer 0 h before and 1, 2, 3, and 4 h after carrageenan injection (24). Acetylsalicylic acid 100 mg/kg was used as a standard drug (18). The percentage inhibition of inflammation was calculated for each group with respect to its vehicle-treated control group using the following relationship (24).

$\mathrm{Inhibition}\left(\%\right)=\frac{(\mathrm{Vt}-\mathrm{Vo})\mathrm{control}-(\mathrm{Vt}-\mathrm{Vo})\mathrm{treated}}{(\mathrm{Vt}-\mathrm{Vo})\mathrm{control}}\times 100$

 Where; V_o_ = right hind paw thickness volume (in mL) before carrageenan injection

V_t_ = right hind paw thickness volume (in mL) after carrageenan injection.

*In-vitro anti-inflammatory activity*

*Hyaluronidase inhibition assay*

Prepared extracts were sent to BioGenics Research and Training Center in Biotechnology (India) for anti-inflammatory testing by the method of hyaluronidase inhibition assay. The assay medium consisting of 5U hyaluronidase (from Sigma–Aldrich, Bangalore) in 100 μL of 20 mM sodium phosphate buffer (pH 7.0) with 77 mM sodium chloride, 0.01% BSA was pre-incubated with different concentrations (10, 50, and 100 μg/mL) of the test extracts and standard drug (Indomethacin) for 15 min at 37 °C. The assay was commenced by adding 100 μL hyaluronic acid (from Sigma-Aldrich, Bangalore; 0.03% in 300 mM sodium phosphate, pH 5.35) to the incubation mixture and incubated for a further 45 min at 37 °C. The undigested hyaluronic acid was precipitated with 1 mL acid albumin solution made up of 0.1% bovine serum albumin in 24 mM sodium acetate and 79 mM acetic acid, (pH 3.75). After standing at room temperature for 10 min, the absorbance of the reaction mixture was measured at 600 nm. The absorbance in the absence of enzyme was used as the reference value for maximum inhibition. The inhibitory activity of each test sample was calculated as the percentage ratio of the absorbance in the presence of test compound *vs.* absorbance in the absence of enzyme. The enzyme activity was checked by control experiment run simultaneously, in which the enzyme was pre-incubated with 5 μL DMSO instead, and followed by the assay procedures described above. The samples were tested in a range of 10 μg-100 μg in the reaction mixture. Indomethacin (Indo) was used as reference standard.

## Results

Powder of dried leaves of *O. fruticosa *(800 g) was macerated and a total of 140 g of a gummy hydroethanolic extract having black color was obtained. The percentage yield of the plant was 17.5%. The percentage yield of the fractions and their physical characteristics is shown below (Table 1). 

*Acute oral toxicity test*

Acute oral toxicity test showed that hydroalcoholic extract of the leaves of *O. fruticosa *has no any signs of toxicities such as loss of appetite, hair erection, lacrimation, tremors, convulsions, salivation, diarrhea, mortality or other signs of overt toxicity during the entire experimental period in mice after oral administration of the extract at a dose level of 2000 mg/kg.

*Analgesic activity*

*Tail immersion test*

In this test, a significant reduction of painful sensation after thermal stimulus to the tail was observed following oral administration of the extract and pethidine intraperitonial (i.p.) compared to negative control (Table 2). Prolongation of reaction time produced by 100 mg/kg of the extract was significantly lower compared to pethidine at all-time points. However, with 200 mg/kg and 400 mg/kg significant difference was noted at 30, 60, 90, 120, as well as 180, 30, and 60 min, respectively, compared to the standard drug. The lowest dose (100 mg/kg) of the extract showed slight analgesic activity following the middle dose (200 mg/kg) which showed the peak analgesia of 7.27 ± 0.48 and 9.50 ± 0.98 s, respectively. The highest dose (400 mg/kg) showed higher analgesic activity with peak analgesia of 12.35 ± 0.41 s. Comparing different doses of the extract revealed that there was a dose-dependent activity. Furthermore, protection against thermal stimuli with 400 mg/kg was significant compared to the other doses of the extract.

*Effects of organic solvent fractions of Otostegia fruticosa on tail immersion test*

The effect of the different fractions of the plant on tail flick latency is shown in Table 3. A considerable analgesic activity was shown with all fractions in comparison with the negative control at all-time points. Prolongation of reaction time produced by hydromethanol and ethyl acetate fractions of the extract were significantly lower compared to pethidine at all-time points. However, with chloroform and butanol fractions significant difference was noted at 30 and 60 min when compared to the standard drug. In this test chloroform and butanol fractions showed better analgesic activity than hydromethanol and ethyl acetate fractions. The chloroform and butanol fractions showed higher analgesic activity which was maximum at 120 min when they showed analgesia of 12.00 ± 0.73 and 11.67 ± 0.56 s, respectively. The hydromethanol and ethyl acetate fractions showed slight analgesic activity which showed a peak at 90 min when they showed analgesia of 6.17 ± 0.21 and 7.22 ± 0.35 s, respectively. Time taken to reach the highest activity for the standard was at 30 min with analgesia of 12.83 ± 0.48 s.

*Writhing test*

As shown in Table 4, mice treated with the standard and 70% ethanol extract of *O. fruticosa*at all doses showed a significant protection (*p* < 0.001) against acetic acid-induced writhing compared to negative control group. Though acetylsalicylic acid produced greater protection, no detectable changes were observed when compared to all doses of the extract. The percentage inhibition observed at a dose of 400 mg/kg (90.9%) and that of acetylsalicylic acid (91.4%) was comparable.

*Effects of organic solvent fractions of Otostegia fruticosa on writhing test*

In this test, the chloroform and butanol fractions showed a better significance reduction in the number of writhes in mice when compared to the negative control, hydromethanol, and ethyl acetate fractions (Table 5). A significant difference (*p < *0.001) was observed with the standard and hydromethanol and ethyl acetate fractions. The hydromethanol fraction showed the lowest (10.2%) inhibition of writhing followed by the ethyl acetate fraction which was 18.2%. The percentage inhibition for the chloroform fraction (85.8%) and the butanol (80.9%) fractions were comparable with the standard (87.6%).

*In-vivo anti-inflammatory activity*

*Carrageenan-induced paw model*

In this model, the higher dose (400 mg/kg) of the extract and the standard drug showed a statistically significant inhibitory effect at 3^rd^ and 4^th^ h on mean increase in paw volume than negative control group (Table 6), while the middle dose (200 mg/kg) of the extract showed a statistically significant inhibitory effect at 4^th^ h. The inhibitory activity produced by 100 mg/kg of the extract was significantly lower compared to the standard at 4^th^ h. Minimum and maximum volume reduction was attained at the 1^st^ and 4^th^ h of the study period, respectively. At the peak of activity (4^th ^h) the percentage inhibition for (100, 200, 400 mg/kg) was 24.2%, 50.4%, and 75.7%, respectively. The inhibition of the standard at the peak of activity (4^th^ h) was 80.6% which was comparable with the higher dose (400 mg/kg) of the extract.

*Effects of organic solvent fractions of Otostegia fruticosa on carrageenan-induced paw model*

In carrageenan-induced paw edema model, the chloroform fraction showed significant inhibitory activity (*p < *0.05 at 3^rd^ and 4^th^ h) and the standard (*p < *0.01at 3^rd^ and *p < *0.001 at 4^th^ h) when compared to the negative control. Maximum and minimum protections from an increase in paw volume were observed at the fourth h and first h respectively, for all fractions and the standard aspirin. At the peak of activity (4^th^ h) the percentage inhibition for hydromethanol, ethyl acetate, chloroform, and butanol fractions was 16.0%, 23.8%, 67.9%, and 51.8%, respectively. The inhibition of the standard was 79.5% (Table 7).

*In-vitro* *anti-inflammatory activity*

*Hyaluronidase inhibition activity evaluation*

In the present study, the *in-vitro *anti-inflammatory properties of extract and organic solvent fractions of the leaf extract of the plant was evaluated using hyaluronidase enzyme inhibition assay. As can be seen in Table 8, all the fractions, crude extract, and standard drug (Indomethacin) exhibited concentration-dependent hyaluronidase inhibition activities in the concentration range (10-100 μg/mL). The crude plant extract and chloroform fraction showed higher activity and the effect of the chloroform fraction was comparable with that of the standard drug, indomethacin.

**Table 1.** Yield and physical properties of solvent fractions of the leaves of O. fruticosa

Fractions	Nature of Extract	Color	Actual Yield (g)	Percentage Yield (w/w%)
**Hexane**	Gummy	Black	1.0	1.5
**Chloroform**	Gummy	Black	36.7	56.5
**Ethyl Acetate**	Gummy	Brown	5.9	9.0
**Buthanol**	Gummy	Brown	4.2	6.4
**Hydromethanol**	Powder	Brown	14.3	22.0

**Table 2:** Effect of 70% ethanol leaf extract of O. fruticosa on tail immersion model in mice

Groups	Latency (s) ± SEM						
	0	30	60	90	120	150	180
**Sunflower oil**	2.91 ± 0.23	2.47 ± 0.17	2.72 ± 0.26	2.55 ± 0.14	2.67 ± 0.21	2.60 ± 0.21	2.33 ± 0.14
**OF 100 mg/kg**	3.23 ± 0.06	4.27 ± 0.43^a2b3c1d3^	4.85 ± 0.36a^2b3c1d3^	5.22 ± 0.47^a2b3c1d3^	6.65 ± 0.78^a3b3d3^	7.27 ± 0.48^a3b2c1d3^	6.78 ± 0.49^a3 b2 d3^
**OF 200 mg/kg**	2.70 ± 0.17	5.92 ± 0.53^a3b3d3^	6.38 ± 0.53^a3 b3d3^	7.12 ± 0.71 ^a3 b3d3^	7.80 ± 0.67^a3 b3d3^	9.50 ± 0.98^a3d2^	8.00 ± 0.95^a3b1d2^
**OF 400 mg/kg**	2.82 ± 0.22	10.58 ± 0.20^a3 b3^	10.97 ± 0.23^a3b3^	11.23 ± 0.21^a3^	11.50 ± 0.33^a3^	12.35 ± 0.41^a3^	11.15 ± 0.29^a3^
**Pethidine 5 mg/kg**	3.40 ± 0.34	14.17 ± 0.09^a3^	13.27 ± 0.11^a3^	12.43 ± 0.16^a3^	11.92 ± 0.03^a3^	10.80 ± 0.21^a3^	10.25 ± 0.21^a3^

Data represent mean ± SEM (n = 6);^ 1^*p* < 0.05, ^2^*p* < 0.01, ^3^*p* < 0.001; ^a^relative to control; ^b^relative to standard; ^c^relative to 200 mg/kg; ^d^relative to 400 mg/kg. OF: 70% ethanol extract of *Otostegia fruticosa.*

**Table 3:** Effect of the organic solvent fractions of 70% ethanol leaf extract of O. fruticosa on tail immersion model in mice

**Groups**	**Latency (s) ± SEM**						
	**0**	**30**	**60**	**90**	**120**	**150**	**180**
Sunflower oil	3.83 ± 0.16	2.63 ± 0.19	2.41 ± 0.18	2.00 ± 0.11	2.70 ± 0.19	2.83 ± 0.21	2.07 ± 0.17
HF400 mg/kg	3.58 ± 0.26	4.33 ± 0.28^b3c3d3^	4.68 ± 0.34^a2b3c3d3^	6.17 ± 0.21^a3b3c3d3^	5.53 ± 0.27^a2b3c3d3^	5.53 ± 0.31^a2b3c3d3^	5.20 ± 0.43^a2b3c3d3^
EF400 mg/kg	8.78 ± 5.45	4.51 ± 0.44^a1b3c3d3^	5.50 ± 0.49^a3b3c3d3^	7.22 ± 0.35^a3b3c2d2^	6.67 ± 0.45 ^a3b2c3d3^	6.43 ± 0.46^a3b3c3d3^	6.20 ± 0.41^a3b3c2d3^
CF400 mg/kg	3.83 ± 0.17	8.00 ± 0.45^a3b3^	9.22 ± 0.47^a3b2^	9.82 ± 0.49^a3^	12.00 ± 0.73 ^a3^	10.08 ± 0.49^a3^	9.17 ± 0.60^a3^
BF400 mg/kg	3.80 ± 0.19	7.93 ± 0.48^a3b3^	9.13 ± 0.44^a3b2^	9.83 ± 0.40^a3^	11.67 ± 0.56 ^a3^	10.33 ± 0.42^a3^	9.58 ± 0.37^a3^
Pethidine 5 mg/kg	4.20 ± 0.35	12.83 ± 0.48^a3^	11.45 ± 0.49^a3^	10.92 ± 0.73^a3^	10.20 ± 0.68^a3^	9.90 ± 0.64^a3^	9.63 ± 0.65^a3^

Data represent mean ± SEM (n = 6); ^1^*p* < 0.05, ^2^*p* < 0.01, ^3^*p* < 0.001; ^a^relative to control; ^b^relative to standard; ^c^relative to BF 400 mg/kg; ^d^relative to HF 400 mg/kg; CF: Chloroform fraction; EF: Ethyl acetate fraction; BF: Buthanol fraction; HF: Hydromethanol fraction; ASA: acetylsalicylic acid.

**Table 4:** Effect of 70% ethanol leaf extract of O. fruticosa on writhing test in mice

Groups	No of writhing ± SEM	Inhibition (%)
**Sunflower oil**	34.83 ± 2.82	
**OF100 mg/kg**	8.00 ± 1.18^a3^	77.0
**OF200 mg/kg**	6.67 ± 0.71^a3^	80.9
**OF400 mg/kg**	3.17 ± 0.48^a3^	90.9
**ASA150 mg/kg**	3.00 ± 0.52^a3^	91.4

The Data represent mean ± SEM (n = 6); ^1^*p* < 0.05, ^2^*p* < 0.01, ^3^*p* < 0.001; ^a^relative to control; ASA: acetylsalicylic acid, OF: 70% ethanol extract of *Otostegia fruticosa. *

**Table 5:** Effect of the organic solvent fractions of 70% ethanol leaf extract of O. fruticosa on writhing test in mice

Groups	No of writhing ± SEM	Inhibition (%)
**Sunflower oil**	37.50 ± 3.67	-
**HF400 mg/kg**	33.67 ± 4.11^b3c3d3^	10.2
**EF400 mg/kg**	30.67 ± 6.59^b3c3d2^	18.2
**CF400 mg/kg**	5.33 ± 0.56^a3^	85.8
**BF400 mg/kg**	7.17 ± 0.70^a3^	80.9
**ASA150 mg/kg**	4.67 ± 0.99^a3^	87.6

The Data represent mean ± SEM (n = 6); ^1^*p < *0.05, ^2^*p < *0.01, ^3^*p < *0.001; ^a^relative to control; ^b^relative to standard; ^c^relative to 400 mg/kg BF; ^d^relative to 400 mg/kg HF; CF: Chloroform fraction; EF: Ethyl acetate fraction; BF: Butanol fraction; HF: Hydromethanol fraction; ASA: acetyl salicylic acid.

**Table 6:** Effect of 70% ethanol leaf extract of O. fruticosa on carrageenan-induced paw edema model

**Groups**	**Mean paw volume ± SEM**				
	**0 h**	**1 h**	**2 h**	**3 h**	**4 h**
Sunflower oil	0.137 ± 0.002	0.178 ± 0.005	0.171 ± 0.004	0.167 ± 0.004	0.160 ± 0.003
OF100 mg/kg	0.136 ± 0.004	0.173 ± 0.002 (7.8)	0.167 ± 0.002 (10.4)	0.161 ± 0.003 (15.4)	0.154 ± 0.003^b2^ (24.2)
OF200 mg/kg	0.135 ± 0.004	0.174 ± 0.005 (5.5)	0.165 ± 0.005 (11.8)	0.158 ± 0.004 (24.5)	0.147 ± 0.003^a1^ (50.4)
OF400 mg/kg	0.138 ± 0.003	0.176 ± 0.004 (3.6)	0.166 ± 0.004 (14.9)	0.152 ± 0.003^a1^ (50.8)	0.143 ± 0.002^a2^ (75.7)
ASA100 mg/kg	0.134 ± 0.002	0.175 ± 0.002 (1.4)	0.164 ± 0.002 (12.4)	0.148 ± 0.001^a2^ (53.8)	0.138 ± 0.002^a3^ (80.6)

The Data represent mean ± SEM (n = 6); ^1^*p < *0.05, ^2^*p < *0.01, ^3^*p < *0.001; ^a^relative to control; ^b^relative to standard; ASA is for acetylsalicylic acid; OF: 70% ethanol extract of *Otostegia fruticosa. *

**Table 7.** Effect of the organic solvent fractions of 70% ethanol leaf extract of O. fruticosa on carrageenan-induced paw edema test in mice

**Groups**	**Mean paw volume ± SEM**				
	**0 h**	**1 h**	**2 h**	**3 h**	**4 h**
Sunflower oil	0.140 ± 0.005	0.173 ± 0.003	0.168 ± 0.002	0.162 ± 0.003	0.156 ± 0.004
HF400 mg/kg	0.132 ± 0.003	0.160 ± 0.004 (3.5%)	0.155 ± 0.004 (5.6%)	0.149 ± 0.003 (7.2%)	0.144 ± 0.002 (16.0%)
EF400 mg/kg	0.133 ± 0.003	0.162 ± 0.003 (1.8%)	0.157 ± 0.002 (2.1%)	0.151 ± 0.002 (8.8%)	0.144 ± 0.002 (23.8%)
CF400 mg/kg	0.135 ± 0.003	0.162 ± 0.003 (5.1%)	0.157 ± 0.003 (12.7%)	0.149 ± 0.003^a1^ (35.0%)	0.140 ± 0.003^a1^ (67.9%)
BF400 mg/kg	0.136 ± 0.003	0.163 ± 0.004 (5.3%)	0.158 ± 0.004 (10.6%)	0.152 ± 0.003 (20.6%)	0.144 ± 0.003 (51.8%)
ASA100 mg/kg	0.132 ± 0.004	0.164 ± 0.004 (3.2%)	0.154 ± 0.005 (13.4%)	0.142 ± 0.004^a2^ (52.6%)	0.134 ± 0.003^a3^ (79.5%)

The Data represent mean ± SEM (n = 6);^ 1^*p < *0.05, ^2^*p < *0.01, ^3^*p < *0.001; ^a^relative to control; CF: Chloroform fraction; EF: Ethyl acetate fraction; BF: Butanol fraction; HF: Hydromethanol fraction; ASA: acetyl salicylic acid.

**Table 8:** Percentage inhibition of hyaluronidase enzyme by test extract and indomethacin

**Concentration**	**Hyaluronidase inhibition%**					
	**BISA**	**MASA**	**SATE**	**SATG**	**CLPO**	**INDOMETACIN**
10 μg	55.17	53.10	56.09	59.20	64.83	61.38
50 μg	60.23	57.36	59.31	70.34	68.74	77.01
100 μg	74.48	59.89	63.56	79.20	85.75	95.52

The data represent BISA: Butanol fraction; MASA: Hydromethanol fraction; SATE: Ethyl acetate fraction; SATG: Crude extract; CLPO: Chloroform fraction.

## Discussion

A more specific model (tail immersion test) based on noxious stimulation of thermon-ociceptors was employed to investigate the central analgesic potential of the extract against this type of pain (25). In this technique, centrally mediated pain is induced at the supraspinal level and has selectivity for centrally acting analgesics. The increase in the reaction time after administration of drug in the tail immersion model shows analgesic activity (6).

In this method, duration of time for peak activity was longer for the crude extract (150 min), fractions (chloroform and butanol) (120 min) and (Hydromethanol and ethyl acetate) (90 min) than for the standard drug (30 min). This time gap may be due to the longer lasting, sustained and pronounced central analgesic effect of the extract at a dose of 400 mg/kg and for the fractions (chloroform and butanol) during the study period as compared to the standard. This might also be the reason for the time lag between drug entering the central compartment and distribution into the target site or formation of active metabolites that are capable of exerting analgesic activity. Better activity of the extract (400 mg/kg) at 150 and 180 min and the fractions (chloroform and butanol) at 120 and 150 min compared to pethidine, suggests that there may be the presence of other numerous active principles in combination that contributes to the analgesic activity of the extract in addition to opioid-like components. 

A decrease in reaction time was observed for the extract, fractions, and standard after peak was achieved. This may be due to the susceptibility of this method to habituation and learning conditions which result in progressive shortening of response reaction time. The lowest (100 mg/kg) and middle (200 mg/kg) doses of the extract and fractions (Hydromethanol and ethyl acetate) showed slight analgesic activity. This might be ascribed to the inability of the secondary metabolites to reach adequate concentrations which are responsible for the antinociceptive activity.

Therefore, the effect of the plant on the tail immersion model confirmed its central effect. This central analgesic activity of *O. fruticosa* is most likely be mediated by central action (spinally and supraspinally) and indicates a codeine-like mechanism by binding to opioid receptors (26). 

The peripheral analgesic activity of the plant was assessed using acetic acid-induced writhling method. It is a well-known technique employed for visceral pain model in rodents. The test is based on the administration of intense chemical stimulus that provokes a nociceptive response of small duration (23). The abdominal constriction accompanied by movements of the hind paws response induced by intraperitoneal injection of acetic acid is a sensitive procedure to establish peripherally acting analgesics. This response is thought to involve local peritoneal receptors. Acetic acid has been found to cause an increase in peritoneal fluid levels of prostaglandins (PGE2 and PGF2), hence causing inflammatory pain by inducing capillary permeability (27). 

In the writhing test, unlike the tail immersion test, 100 mg/kg of the extract showed significant analgesic activity. This is because the method is sensitive and reliable to detect anti-nociceptive effects of compounds at lower doses (27). At the middle and higher doses (200 mg/kg and 400 mg/kg) the extract had comparable activity with the standard indicating an increase in the concentration of phytoconstituents that possess analgesic activity with increasing dose. The fractions of *O. fruticosa *also showed a varying degree of protection in response to intraperitoneal acetic acid administration. The hydromethanol and ethyl acetate fractions did not show significant protections, which might be due to lower concentrations of active secondary metabolites. But the chloroform and butanol fractions showed significant reductions (*p < *0.001) which was comparable with the standard aspirin.

These findings suggest that this plant has peripheral analgesic activity and its mechanisms of action may be mediated through inhibition of local peritoneal receptors which may be due to the involvement of cyclooxygenase inhibition potential or due to the interference of its active principle(s) with the release of pain mediators (8). Since this method is a non-selective method (28), it is possible that other mechanisms besides PG inhibition could play a role in the analgesic action of the extract. Since the chloroform and butanol fractions showed greater protection of writhing than those of hydromethanol and ethyl acetate fractions, so it can be assumed that secondary metabolites which are nonpolar and/or polar might be responsible for the activity. 

From this study, it seems that the analgesic effects produced by the extract may be attributed individually or collectively to the secondary metabolites present in the crude extract. Preliminary phytochemical screening of the 70% ethanol extract of this plant revealed the presence of flavonoids, tannins, phenols, and anthraquinones. These secondary metabolites have been reported to exert analgesic and anti-inflammatory activities. Flavonoids are widely shown to target prostaglandins which are involved in the pain perception through moderating opioidergic mechanism (6, 28 and 29-34). The analgesic activity of this medicinal plant may be due to the interference of its active principles with the release of pain mediators which can be attributed to the above class of natural products. 

Carrageenan-induced hind paw edema model was used to evaluate the anti-inflammatory activity of the extract. Freund’s adjuvant, dextran, cotton pellet granuloma and formalin are other inflammatory models (35). Carrageenan-induced hind paw edema is the standard experimental model of acute inflammation; it is the phlogistic agent of choice for testing anti-inflammatory drug as it is not known to be antigenic and is devoid of apparent systemic effects. Moreover, the experimental model exhibits a high degree of reproducibility (36). This model is a predictive test for anti-inflammatory agents which act by inhibiting mediators of acute inflammation (29). Carrageenan is a natural carbohydrate derived from a number of seaweeds of the class Rhodophyceae (35). Its induction of inflammation involves three distinct phases of mediators release including histamine and serotonin in the first phase which occurs between 0 and 1.5 h of carrageenan injection, bradykinins in the second phase (1.5-2.5 h) and prostaglandins in the third phase which occurs from 2.5 to 6 h post-carrageenan injection (30).

The crude extract at a dose of 400 mg/kg and the chloroform fraction showed the highest anti-inflammatory activity, and the lowest inhibitory activity was seen at the lower dose (100 mg/kg) and the hydromethanol fraction. The extract at the three dose levels and all fractions achieved maximum anti-inflammatory activity at the 4^th^ h indicating that it contains bioactive constituents which are active against the release of prostaglandins. The higher anti-inflammatory activity of the extract at the 400 mg/kg dose level than the lower dose levels and higher anti-inflammatory activity of the chloroform fraction than those of the hydromethanol and ethyl acetate fractions was observed. 

During the early phase of post-carrageenan injection, the crude extract and all fractions were less effective in inhibiting the carrageenan-induced edema. This can be described that the anti-inflammatory activity is less likely to be due to inhibition of histamine and serotonin release (28). 

Flavonoids are known to target prostaglandins which are involved in the late phase of acute inflammation and pain perception and tannins act as primary antioxidants or free radical scavengers (29-35). Polyphenols are also a major group of compounds that act as primary antioxidants or free radical scavengers. They exert their anti-inflammatory properties through inhibition of the production of inflammatory cytokines and chemokines and suppressing the activity of cyclooxygenase (COX) and inducible nitric oxide synthase (iNOS) thus decreasing the production of ROS and RNS and then act as primary antioxidants or free radical scavengers (27, 36). Triterpenoids inhibit the production of prostaglandins and also suppresses the function of macrophages and neutrophils (35). These constituents could also be responsible for the anti-inflammatory potential of the studied plant. Therefore, the anti-inflammatory action of the crude extract and fractions of *O. fruticosa* observed in carrageenan-induced paw edema model could possibly be due to the presence of flavonoids, tannins, polyphenols, and terpenoids acting either individually or synergistically. 

Hyaluronan (also called hyaluronic acid or hyaluronate or HA) is a lipopolysaccharide, which has important biological functions in bacteria and higher animals including humans. It is naturally synthesized by hyaluronan synthases and degraded by a family of enzymes called hyaluronidases (37, 38). Hyaluronidase hydrolyzes HA in the extracellular matrix during tissue remodelling, and up-regulation of hyaluronidase activity occurs in chronic inflammatory conditions (39). Hyaluronidase inhibitors are recommended to have a beneficial role in the prevention and treatment of inflammatory disorders (40). Therefore, the hyaluronidase enzyme inhibition activity had shown by the crude and organic solvent fractions of *O. fruticosa*: could partially contribute to the traditional use against inflammatory related disorders.

## Conclusion

The present study attempted to evaluate the analgesic and anti-inflammatory activity of the leaf extract of *O. fruticosa* (Forssk.). The ability of the extract to prolong the reaction latency to thermally induced pain, inhibiting the acetic acid-induced writhling, carrageenan-induced inflammation, and hyaluronidase inhibition activity confirms the analgesic and anti-inflammatory activities of the extract. The analgesic and anti-inflammatory effects of the plant could be through inhibition of the cell mediators such as prostaglandins and also central and other peripheral inhibitory mechanisms. Therefore, the results from this study support the traditional use of this plant in relieving pain and inflammatory conditions.
